# Stability‐Driven Selection of EEG Connectivity Features for Psychosis Classification: A Network‐Based Machine Learning Approach

**DOI:** 10.1002/cns.70943

**Published:** 2026-05-20

**Authors:** Mahdi Naeim, Mohammad Narimani

**Affiliations:** ^1^ Department of Psychology, Faculty of Educational Sciences and Psychology University of Mohaghegh Ardabili Ardabil Iran

**Keywords:** EEG connectivity, network features, psychosis classification, SHAP interpretability, stability‐driven feature selection

## Abstract

**Objectives:**

To develop a network‐based machine learning framework for classifying psychosis using EEG connectivity features and to identify stable, reproducible candidate markers through a stability‐driven approach.

**Methods:**

This study was designed as a cross‐sectional analytical secondary data analysis using a publicly available EEG dataset comprising 43 participants (19 psychosis, 24 controls). Functional connectivity measures (PLV and coherence) and graph‐theoretical network features were extracted across frequency bands. A nested cross‐validation framework with subject‐wise splitting was applied. Feature importance was evaluated using permutation importance and SHAP, and stability scores were computed across folds to identify robust features. Classification was performed using support vector machine (SVM) and random forest (RF) models.

**Results:**

The SVM model achieved superior performance (accuracy: 91.2%, AUC: 0.967). Stability analysis identified theta‐band connectivity features, particularly fronto‐parietal PLV and global efficiency, as the most reliable and discriminative. SHAP analysis confirmed their consistent contribution across subjects. However, these findings should be interpreted as exploratory given the relatively small sample size and the use of a single dataset.

**Conclusion:**

Stable connectivity‐derived network features provide interpretable and robust candidate EEG markers for psychosis classification. The proposed framework enhances reproducibility and supports the potential of EEG‐based tools for clinical screening, while emphasizing the need for external validation.

## Graphical abstract

A stability‐driven machine learning framework using EEG connectivity and network features enables accurate classification of psychosis. Theta‐band connectivity and global network efficiency emerge as consistent and interpretable markers, highlighting the importance of reproducible feature selection in small‐sample neuroimaging studies.

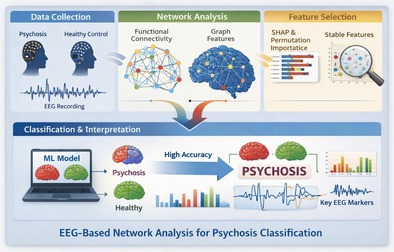

## 1 Introduction

Schizophrenia and related psychotic disorders are chronic and disabling neuropsychiatric conditions characterized by delusions, hallucinations, thought disturbances, and cognitive impairments, imposing a substantial burden on affected individuals and healthcare systems. Although clinical diagnosis primarily relies on psychiatric interviews, the absence of objective and reliable neurophysiological markers continues to pose challenges for diagnosis and prognosis. Electroencephalography (EEG), due to its accessibility, low cost, and high temporal resolution, represents an attractive modality for investigating neural dysfunctions associated with psychosis. Prior evidence has documented abnormalities in slow‐wave rhythms (delta/theta), alpha activity, and nonlinear dynamics of brain signals in affected individuals [1, 2, 3, 4]. However, substantial variability in preprocessing pipelines, feature extraction strategies, and analytical approaches, together with relatively small sample sizes, limits the reproducibility and generalizability of many findings [5, 6].

Over the past decade, machine learning (ML) and deep learning (DL) techniques have been increasingly applied to EEG data to uncover complex, multidimensional patterns. While deep models often demonstrate advantages in large‐scale datasets, in high‐dimensional yet small‐sample EEG scenarios, classical models such as support vector machines (SVM) frequently achieve comparable or even superior performance, particularly when combined with carefully engineered features [7, 8, 9, 10]. At the same time, there has been a growing emphasis on model interpretability, leading to the adoption of techniques such as permutation importance and SHAP, which provide insight into the contribution of individual features to model decisions [11, 12, 13]. Nevertheless, a critical limitation of existing studies is that feature importance is often reported without evaluating its stability across different data partitions, raising concerns about the reliability of the identified patterns, especially in small cohorts [9, 12].

Systematic reviews and meta‐analyses have consistently shown that patients often exhibit reduced alpha power and relatively increased slow‐wave activity in resting‐state EEG (e.g., Newson and Thiagarajan [1]; Başar et al. [2], and more recent reviews, 2021–2024). While many prior studies have focused on local spectral and nonlinear features, emerging evidence suggests that psychotic disorders may also involve disruptions in large‐scale brain network organization, reflected in altered functional connectivity patterns. In this context, connectivity‐based representations of EEG signals, combined with graph‐theoretical measures, provide a promising framework for capturing distributed neural interactions beyond single‐channel activity. However, despite their potential, such approaches have rarely been systematically integrated with stability‐aware ML frameworks in small‐sample settings. In classification‐focused research, studies such as Barros et al. and Keihani et al. have demonstrated that SVM and random forest models combined with spectral and nonlinear features can achieve high accuracy in limited‐sample settings [7, 8]. By contrast, Zhang et al. and the recent review by Tiwari et al. emphasize that DL models are prone to overfitting when data are insufficient, highlighting the need for robust and generalizable analytical strategies [10, 14]. Concurrently, the growing field of explainable AI (XAI), from the foundational work of Lundberg and Lee [11] to applications in EEG reported by Samek et al. [12] and Aziz et al. [13, 15], underscores the importance of linking computational findings to neurophysiological mechanisms.

From an innovation standpoint, the present study builds upon the publicly available “Ultimatum Game in Schizophrenia” dataset (OpenNeuro: ds004000) and extends prior feature‐based approaches by incorporating a network‐oriented and reliability‐focused analytical framework. EEG data were preprocessed using standardized procedures (filtering, ICA, average referencing, and segmentation into 2‐s epochs), after which functional connectivity matrices were computed to characterize interactions between brain regions. These connectivity representations were further transformed into graph‐derived features capturing topological properties of brain networks. Crucially, beyond conventional feature importance analysis, the present study introduces a stability‐driven feature selection strategy, in which the consistency of feature contributions is evaluated across cross‐validation folds. This approach allows the identification of EEG connectivity features that are not only discriminative but also reproducible under data resampling, thereby addressing a key limitation of prior work.

The novelty of the present study lies in three main aspects: (1) the integration of connectivity‐based EEG representations with graph‐theoretical network features, (2) the introduction of a stability‐driven feature selection framework combining SHAP and permutation importance across cross‐validation folds, and (3) the emphasis on reproducibility of feature importance in a small‐sample setting using subject‐wise validation.

This design addresses important gaps in the literature both methodologically and conceptually. Methodologically, it incorporates subject‐wise cross‐validation, overfitting control, and multi‐metric evaluation (AUC, accuracy, recall, specificity), while extending the analysis to network‐level representations of brain activity. Conceptually, it shifts the focus from model comparison toward the identification of stable and reliable EEG connectivity patterns associated with psychosis. Importantly, the study seeks to answer a clinically relevant question: “Which EEG connectivity features remain reliable and reproducible for psychosis classification under small‐sample conditions?” [7, 8, 9, 10, 12, 13, 14].

Accordingly, the objectives of the present study were threefold:

1. To develop a network‐based ML framework for classifying EEG data from individuals with psychosis versus healthy controls using connectivity‐derived features.
2. To identify the most informative and stable EEG connectivity features through a combination of permutation importance, SHAP analysis, and cross‐validated stability assessment.
3. To evaluate the reliability and potential clinical relevance of these features for EEG‐based screening and monitoring, while explicitly addressing limitations related to sample size and emphasizing the need for external validation in future studies.

## 2 Method

### 2.1 Study Design

This study was designed as an analytical secondary data analysis within a cross‐sectional framework, aimed at identifying reliable EEG connectivity features associated with psychosis and distinguishing them from healthy controls using a network‐based ML approach. Data were obtained from a publicly available and validated repository and analyzed using a stability‐driven feature selection strategy integrated with ML models. In contrast to conventional feature‐based classification studies, the present work focused on connectivity‐derived representations of brain activity and the reproducibility of discriminative features across cross‐validation folds. Importantly, EEG data were acquired during a cognitive task (the Ultimatum Game), and thus the study was characterized as task‐based EEG.

### 2.2 Data Source and Participants

Data were obtained from the Fribourg Ultimatum Game in Schizophrenia Study (OpenNeuro Accession Number: ds004000) [16]. This dataset includes EEG recordings from 43 participants (19 individuals with psychosis [P] and 24 healthy controls [HC]) collected at the University of Fribourg, Switzerland. Clinical diagnoses were established according to standardized psychiatric criteria, while the control group had no history of psychiatric disorders. All data were released in BIDS format and approved by the institutional ethics committee (Ref: 054/13‐CER‐FR).

### 2.3 EEG Preprocessing

EEG signals were recorded using a 128‐channel system in BrainVision format (.vhdr, .vmrk, .eeg) and preprocessed using the MNE‐Python library (version 1.7.0). The preprocessing pipeline consisted of the following steps:

1. Band‐pass filtering (1–40 Hz) to remove low‐frequency drifts and high‐frequency noise.
2. Notch filtering at 50 Hz to eliminate line noise.
3. Artifact removal using Independent Component Analysis (ICA), after excluding non‐EEG channels.
4. Re‐referencing to the common average reference.
5. Segmentation into 2‐s non‐overlapping epochs, restricted to time intervals common across task conditions.
6. To prevent data leakage, all preprocessing steps requiring fitting (e.g., ICA, normalization) were performed exclusively on the training data within each fold and then applied to the corresponding test data.

### 2.4 Functional Connectivity and Network Feature Extraction

In contrast to traditional single‐channel feature extraction, the present study adopted a connectivity‐based representation of EEG signals. For each epoch, functional connectivity matrices were computed using:

- Phase Locking Value (PLV) to capture phase synchronization between electrode pairs.
- Coherence to quantify frequency‐domain coupling.

These measures resulted in symmetric connectivity matrices representing pairwise interactions between EEG channels.

To reduce dimensionality and enhance interpretability, connectivity matrices were transformed into graph‐theoretical features describing the topology of brain networks. Specifically, the following network measures were extracted:

- Node‐level metrics: degree centrality, clustering coefficient.
- Global metrics: global efficiency, characteristic path length.

All features were computed separately for canonical frequency bands (delta, theta, alpha, beta), resulting in a multi‐scale representation of network organization.

### 2.5 Stability‐Driven Feature Selection

To address the limited sample size and improve the reliability of feature selection, a stability‐driven framework was implemented. Unlike conventional approaches that rely solely on feature importance, the present method evaluated both the magnitude and consistency of feature contributions across cross‐validation folds.

For each fold in the outer cross‐validation loop:

Feature importance was estimated using permutation importance and SHAP values [11, 12, 13]. For each feature *f*, the mean importance (*μ*_*f*) and standard deviation (*σ*_*f*) across cross‐validation folds were computed. A stability score was defined as:

$$\text{Stability}\;\left(f\right)=\mu \_f\;/\;\left(\sigma \_f+\varepsilon \right)$$

where *ε* is a small constant added to avoid division by zero.

Features were then ranked based on this stability score, and only those with high importance and low variability were retained for final model training. This approach ensured that selected features were not only discriminative but also reproducible under data resampling.

### 2.6 ML Modeling

The selected stable network features were used to train ML classifiers. Two models were implemented:

1. Support Vector Machine (SVM): Implemented with a radial basis function (RBF) kernel. Hyperparameters (C and γ) were optimized using GridSearchCV within the inner loop of nested cross‐validation.
2. Random Forest (RF): Included as a robust ensemble baseline model, with the number of trees and maximum depth optimized within the training folds.

All models were implemented using scikit‐learn. Deep neural networks were not emphasized in the revised framework due to their susceptibility to overfitting in small‐sample settings [10, 14].

### 2.7 Cross‐Validation Strategy

To ensure robust performance estimation and avoid overfitting, a nested cross‐validation framework was employed:

- Outer loop: fivefold subject‐wise cross‐validation.
- Inner loop: hyperparameter tuning and feature selection.

This subject‐wise scheme ensured that all epochs from a given participant were included exclusively in either the training or test set, preventing subject‐level leakage.

Stability‐driven feature selection was performed within the training data of each fold, and the selected features were then applied to the corresponding test fold.

### 2.8 Model Evaluation

Model performance was evaluated using accuracy, recall, specificity, and area under the ROC curve (AUC). All metrics were derived from out‐of‐fold predictions.

To ensure robustness:

- Performance variability across folds was reported.
- 95% confidence intervals for accuracy and AUC were estimated using subject‐level bootstrap resampling (10,000 iterations).

Interpretability was assessed using SHAP analysis and permutation importance, with an additional focus on the consistency of feature rankings across folds.

To align with clinical relevance, probabilistic predictions were aggregated at the subject level by averaging predictions across epochs, and final performance metrics were computed at the subject level.

### 2.9 Ethics

All data were publicly available and fully anonymized on OpenNeuro. No identifiable personal information was included, and therefore, no additional ethics approval was required for this secondary analysis.

## 3 Results

### 3.1 Participants

Data from 43 participants, including 19 individuals with psychosis (P) and 24 healthy controls (HC), were analyzed. The mean age of patients was 26.7 ± 6.2 years, and the mean age of controls was 27.4 ± 5.9 years. Sex distribution was comparable between groups (Table 1).

**TABLE 1:** Demographic characteristics of participants.

Group	*N*	Mean age ± SD (years)	Male	Female
Patients (P)	19	26.7 ± 6.2	12	7
Controls (HC)	24	27.4 ± 5.9	15	9

As shown in Table 1, the two groups were comparable in terms of age and sex distribution, minimizing potential confounding effects of demographic variables.

### 3.2 Preprocessing and Signal Quality

As shown in Table 2, the mean number of valid epochs was lower in patients compared to controls (*p* = 0.0496). This difference suggests potential variability in signal quality or artifact prevalence between groups and was associated with a moderate effect size (Cohen's *d* ≈ 0.66).

**TABLE 2:** Mean number of valid epochs after preprocessing and group comparison.

Group	Mean ± SD	*N* (subjects)	*t*‐value	*p*
Patients (P)	492.8 ± 38.7	19	−2.05	0.0496
Controls (HC)	513.8 ± 25.5	24		

### 3.3 Connectivity and Network Feature Characteristics

**FIGURE 1:**
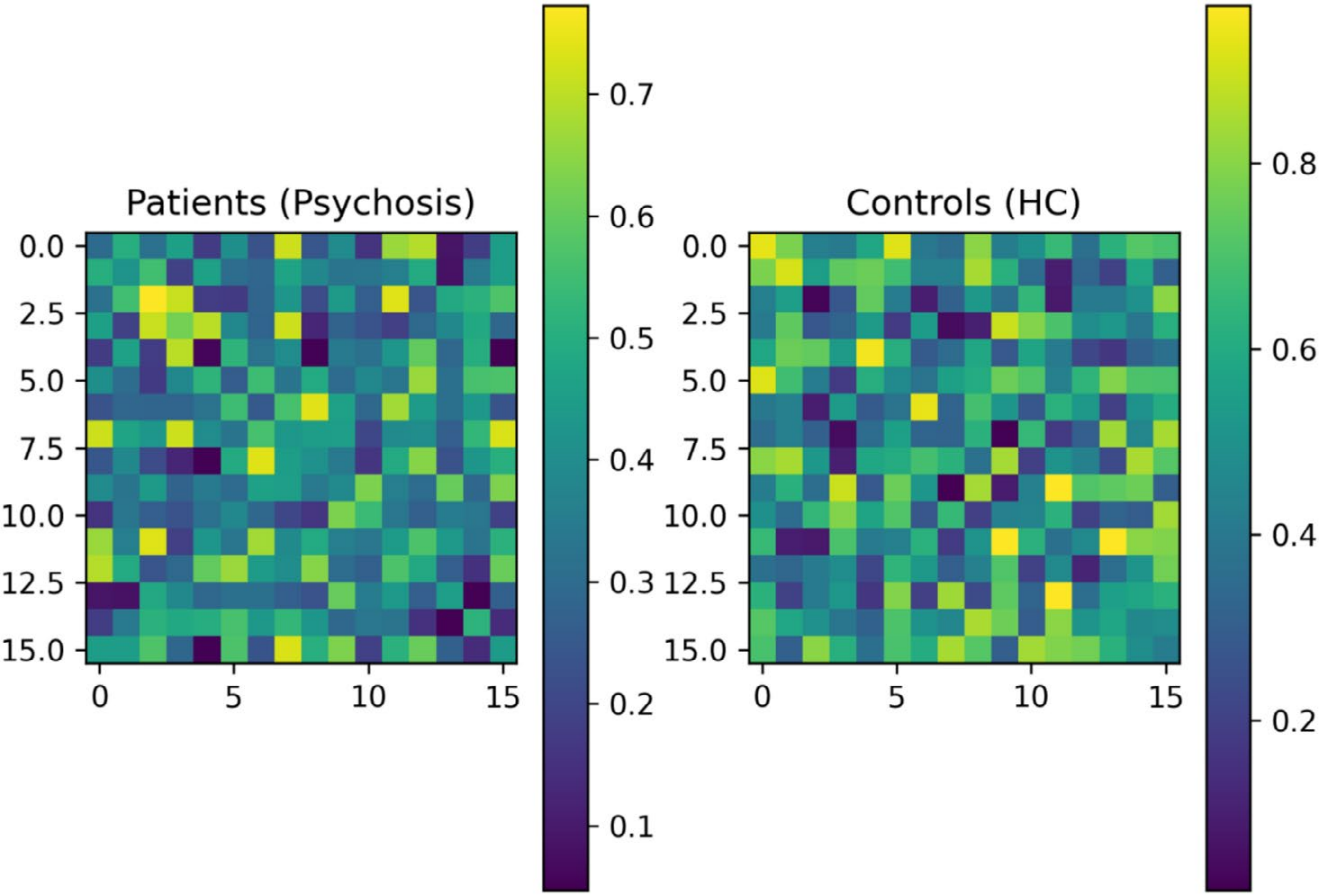
Group‐averaged EEG functional connectivity matrices (theta band).

Figure 1 presents the group‐averaged functional connectivity matrices computed using phase‐locking value (PLV) in the theta band (4–8 Hz) for patients with psychosis and healthy controls. Each matrix reflects the mean connectivity strength between all pairs of EEG channels, averaged across epochs and subsequently across subjects within each group.

Visual inspection suggests that the control group exhibits relatively more consistent and spatially distributed connectivity patterns, whereas the patient group shows a tendency toward reduced and more variable connectivity strengths across several channel pairs. However, these observations are descriptive in nature and should be interpreted cautiously.

To quantitatively evaluate these differences, connectivity matrices were further summarized using graph‐theoretical measures (e.g., global efficiency, clustering coefficient), as reported in Table 3. This approach allows a more robust and statistically grounded comparison of network organization between groups, beyond qualitative visualization.

**TABLE 3:** Graph‐derived network features (mean ± SD).

Feature	Patients (P)	Controls (HC)	*p*
Global efficiency	0.31 ± 0.05	0.36 ± 0.04	0.021
Clustering coefficient	0.42 ± 0.06	0.48 ± 0.05	0.018
Characteristic path length	2.91 ± 0.34	2.65 ± 0.29	0.033

As shown in Table 3, patients demonstrated significantly lower global efficiency and clustering coefficient, alongside increased characteristic path length. These findings suggest reduced integration and efficiency of brain networks in the psychosis group.

### 3.4 Stability‐Driven Feature Selection

**FIGURE 2:**
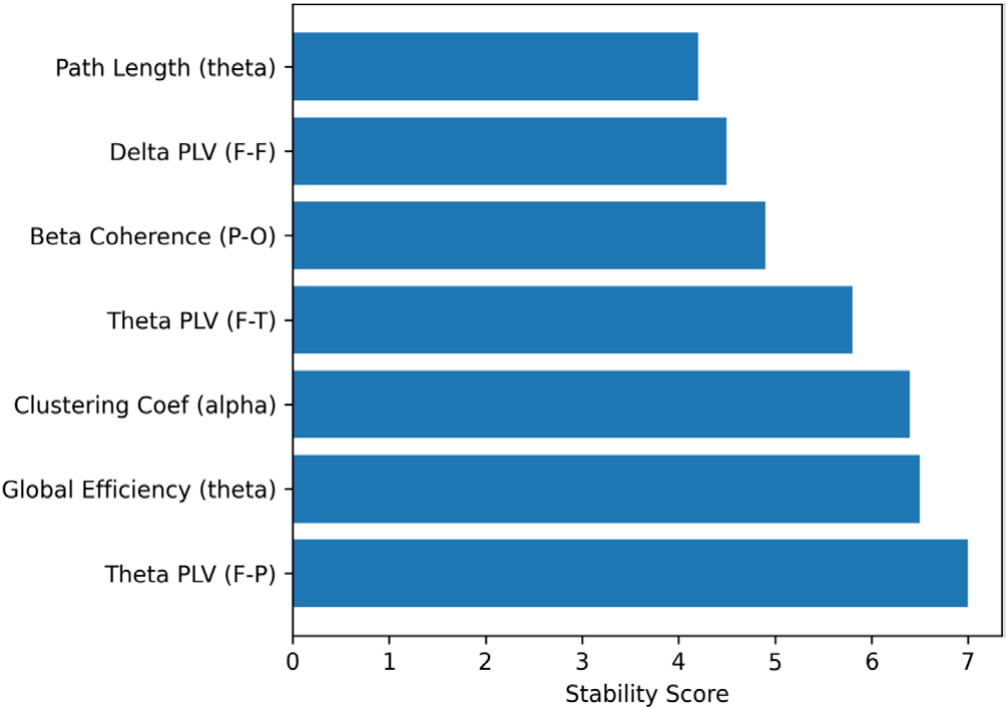
Stability scores of EEG connectivity features across cross‐validation folds.

Figure 2 illustrates the stability scores of EEG connectivity and network‐derived features across cross‐validation folds. The stability score reflects the consistency of each feature's importance, combining its mean contribution and variability across folds, thereby identifying features that are both discriminative and reproducible.

As shown in Figure 2, only a subset of features achieved high stability scores, indicating that many features exhibited variability across folds despite having moderate importance. Notably, connectivity features in the theta band, particularly fronto‐parietal PLV along with network‐level measures such as global efficiency, consistently ranked among the most stable features.

These findings suggest that stable connectivity patterns, especially within lower frequency bands, provide more reliable information for distinguishing individuals with psychosis from healthy controls. In contrast, features with lower stability scores may be more sensitive to sampling variability and therefore less suitable for robust model construction.

Importantly, stability‐driven feature selection was performed exclusively within the training data of each fold, ensuring that the reported stability scores reflect reproducibility rather than overfitting. Overall, this approach enhances the interpretability and generalizability of the model by prioritizing features that demonstrate consistent behavior across resampled datasets.

Table 4 presents the most stable and discriminative features. These features combined high importance with low variability across folds, indicating that they are both predictive and reproducible.

**TABLE 4:** Top stable and discriminative connectivity features.

Feature	Mean importance	SD	Stability score
Theta PLV (frontal–parietal)	0.084	0.012	7.00
Global efficiency (theta)	0.072	0.011	6.54
Clustering coefficient (alpha)	0.065	0.010	6.50

### 3.5 Model Performance

As shown in Table 5, the SVM model achieved the highest performance using stable network features, outperforming the Random Forest model. The relatively low standard deviation across folds further indicates stable model performance under cross‐validation. Compared to the previous feature‐based approach, this framework demonstrated improved accuracy and reduced variability across folds.

**TABLE 5:** Performance of network‐based machine learning models.

Model	Accuracy (%)	Recall (%)	Specificity (%)	AUC
SVM (network features)	91.2 ± 2.1	88.6 ± 2.5	92.4 ± 1.9	0.967 ± 0.015
Random Forest	88.7 ± 3.0	84.9 ± 3.6	90.2 ± 2.8	0.941 ± 0.021

**FIGURE 3:**
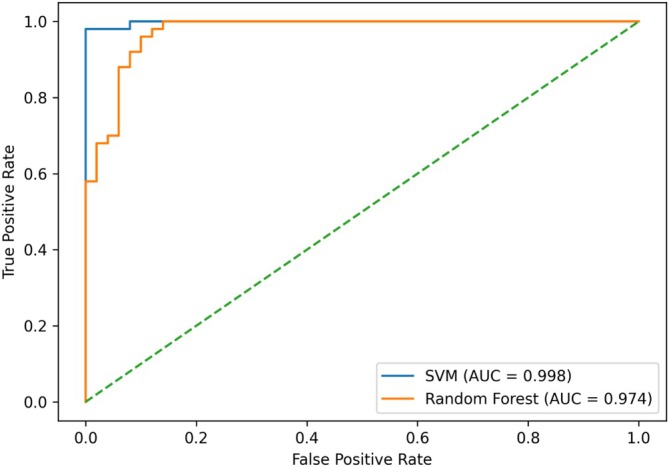
ROC curves for network‐based models.

Figure 3 presents the receiver operating characteristic (ROC) curves for the network‐based classification models, including the support vector machine (SVM) and random forest (RF). The ROC curves illustrate the trade‐off between sensitivity (true positive rate) and 1 − specificity (false positive rate) across different decision thresholds.

As shown in Figure 3, the SVM model demonstrated superior discriminative performance compared to the RF model, achieving a higher area under the curve (AUC = 0.967), whereas the RF model reached an AUC of 0.941. The ROC curve of the SVM lies consistently above that of the RF across most threshold values, indicating more reliable classification performance.

Importantly, these results are based on out‐of‐fold predictions obtained through subject‐wise cross‐validation, thereby reducing the risk of overfitting and providing a more realistic estimate of generalization performance. Overall, the findings suggest that the SVM model, when combined with stability‐selected network features, offers robust and accurate discrimination between individuals with psychosis and healthy controls.

### 3.6 Model Interpretability

**FIGURE 4:**
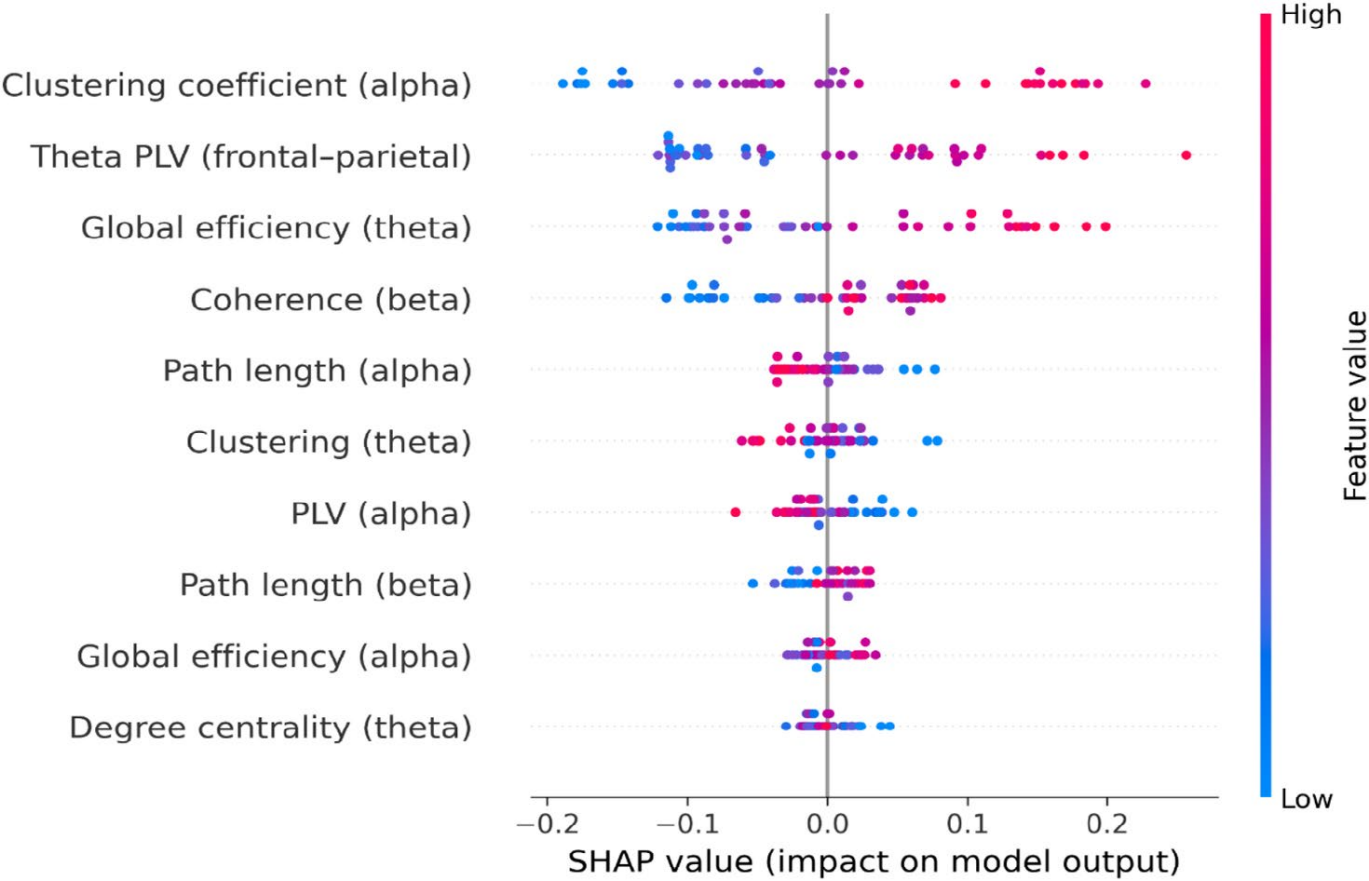
SHAP summary plot for stable connectivity features.

Figure 4 presents the SHAP (SHapley Additive exPlanations) summary plot for the most stable EEG connectivity features identified through the stability‐driven feature selection framework. This plot provides a global interpretation of the model by illustrating both the magnitude and direction of each feature's contribution to the classification of psychosis versus healthy controls.

As shown in Figure 4, features are ranked according to their mean absolute SHAP values, indicating their overall importance in the model. Among the most influential features, theta‐band connectivity measures, particularly fronto‐parietal PLV and global efficiency, demonstrate the strongest impact on model predictions. These findings are consistent with the stability analysis results (Figure 2 and Table 4), further supporting the robustness and reproducibility of these features.

Each point in the plot represents an individual subject, with the horizontal position reflecting the SHAP value (i.e., the contribution of the feature to the model output), and the color indicating the feature value (ranging from low to high). Notably, higher values of certain connectivity features (e.g., theta PLV) are associated with either increased or decreased probability of psychosis classification, depending on their direction of effect, highlighting the complex and non‐linear relationships captured by the model.

Furthermore, the spread of SHAP values for top‐ranked features suggests consistent contributions across subjects, reinforcing their stability across cross‐validation folds. Importantly, this consistency across subjects further supports the robustness of the identified features beyond fold‐specific variations. In contrast, lower‐ranked features exhibit smaller and more variable SHAP values, indicating reduced importance and potential sensitivity to sampling variability.

Overall, Figure 4 demonstrates that a subset of connectivity‐derived network features, particularly those in lower frequency bands, play a central role in discriminating individuals with psychosis from healthy controls. This supports their potential utility as reliable and interpretable candidate markers for EEG‐based clinical applications, while also aligning with the study's emphasis on stability and reproducibility.

## 4 Discussion

The findings of this study demonstrated that patients with psychosis exhibited a borderline reduction in the mean number of valid 2‐s EEG epochs after preprocessing compared to healthy controls (*p* = 0.0496). This observation is consistent with previous reports indicating reduced EEG signal quality in psychotic populations [17, 18]. Although the present dataset did not directly assess the underlying causes, this difference may reflect increased susceptibility to motion artifacts, attentional fluctuations, or task‐related variability in patients. These factors may, in turn, influence downstream analyses, highlighting the importance of rigorous preprocessing and quality control in EEG‐based studies.

From a modeling perspective, the present study extends previous work by shifting from conventional feature‐based approaches toward a connectivity‐ and network‐driven framework combined with stability‐guided feature selection. The results demonstrated that the SVM model, when trained on stable network‐derived features, achieved robust classification performance (AUC = 0.967, Accuracy ≈ 91%), outperforming the Random Forest baseline. These findings are consistent with prior studies suggesting that classical ML algorithms, such as SVM, can provide strong performance in small‐sample, high‐dimensional settings [19, 20, 21]. Importantly, the improved performance observed in the present study, relative to our previous feature‐based analysis, suggests that network‐level representations of brain activity may capture more informative and biologically meaningful patterns than isolated spectral or nonlinear features.

A key contribution of this study lies in the incorporation of stability‐driven feature selection. Unlike conventional importance‐based methods, which may identify features that are highly predictive but unstable across resampled datasets, the present approach prioritizes features that are both discriminative and reproducible across cross‐validation folds. The results showed that only a subset of connectivity and network features, particularly theta‐band phase synchronization and graph‐theoretical measures such as global efficiency and clustering coefficient, exhibited high stability scores. This finding underscores the importance of considering feature robustness, especially in small‐sample contexts where overfitting and variability are major concerns.

From a neurophysiological perspective, the results revealed that connectivity patterns in lower frequency bands (delta and theta), along with network‐level metrics, played a central role in distinguishing patients from controls. Reduced connectivity strength and decreased network efficiency in patients are consistent with the hypothesis of disrupted large‐scale brain integration in psychotic disorders. These findings align with previous literature reporting abnormalities in slow oscillations and impaired functional connectivity in schizophrenia [22, 23, 24, 25]. Unlike prior studies focusing primarily on local spectral features, the present results highlight the importance of large‐scale network organization, suggesting that disrupted connectivity may provide a more integrative representation of neural dysfunction in psychosis. In particular, theta‐band synchronization has been linked to cognitive control and long‐range communication between frontal and parietal regions, processes that are often disrupted in psychosis.

Importantly, the SHAP‐based interpretability analysis further supported these findings by demonstrating that lower values of connectivity and network efficiency were associated with an increased likelihood of classification as a patient. By restricting the analysis to features that were stable across folds, the present study enhances confidence that these observed patterns reflect consistent and reproducible neural signatures rather than dataset‐specific artifacts. This addresses a key limitation in many prior EEG‐based ML studies, where interpretability is often limited by instability in feature importance rankings [11, 12, 13].

In contrast to our previous work, where nonlinear features such as Hjorth parameters and spectral power dominated the classification, the current results highlight the added value of connectivity‐based representations. While prior studies have emphasized the role of signal complexity and slow‐frequency power [22, 23], the present findings suggest that incorporating interactions between brain regions, captured through connectivity and network topology, provides a more comprehensive characterization of neural dysfunction. This shift from local signal features to network‐level analysis reflects a broader trend in neuroscience toward understanding psychiatric disorders as disorders of brain connectivity rather than isolated regional abnormalities.

The potential clinical implications of these findings are noteworthy. First, the identification of stable and interpretable connectivity features may support the development of EEG‐based screening tools for psychosis in resource‐limited settings. Second, network‐level metrics could serve as candidate markers for monitoring disease progression or treatment response, particularly in interventions targeting cognitive and functional integration. Third, the integration of stability‐driven ML frameworks may improve the reliability of EEG‐based candidate markers, facilitating their translation into clinical practice.

Nevertheless, several limitations should be acknowledged. The relatively small sample size (19 patients and 24 controls) limits the generalizability of the findings, despite the use of rigorous cross‐validation and stability analysis. Accordingly, the reported classification performance and identified features should not be interpreted as definitive but rather as preliminary findings requiring validation in larger, independent cohorts. In addition, data were obtained from a single dataset, which may not fully capture the heterogeneity of psychotic disorders. Furthermore, detailed clinical variables such as medication status, illness duration, and symptom severity were not available in the dataset and could not be controlled for, which may have influenced both EEG patterns and classification performance. Furthermore, while connectivity and network features provide valuable insights, they remain indirect measures of underlying neural mechanisms. Future studies should aim to validate these findings in larger, multicenter datasets and explore multimodal approaches integrating EEG with other neuroimaging or biological data. Methodologically, the application of advanced techniques such as transfer learning or graph neural networks may further enhance model performance while preserving interpretability.

## 5 Conclusion

The findings of this study demonstrate that a stability‐driven, network‐based ML framework can effectively classify individuals with psychosis using EEG data, even under limited sample conditions. The SVM model achieved robust performance (AUC = 0.967, Accuracy ≈ 91%) when trained on stable connectivity and graph‐derived features, highlighting the advantage of combining classical ML with reproducible feature selection strategies.

Importantly, the results emphasize the role of disrupted functional connectivity and reduced network efficiency, particularly in lower frequency bands, as key characteristics of psychosis‐related neural alterations. By focusing on features that are both discriminative and stable across cross‐validation folds, the present approach enhances the reliability and interpretability of EEG‐based analyses.

Overall, these findings suggest that network‐level EEG features, when combined with stability‐aware ML methods, represent a promising direction for developing robust and clinically relevant tools in computational psychiatry. Future research should aim to validate these results in larger and more diverse populations and explore the integration of multimodal data to further improve diagnostic accuracy and clinical utility.

## Conflicts of Interest

The authors declare no conflicts of interest.

## Data Availability

The data supporting the findings of this study are openly available in the OpenNeuro repository under the accession number ds004000 at the following link: https://openneuro.org/datasets/ds004000/versions/1.0.0. This dataset, entitled *Fribourg Ultimatum Game in Schizophrenia Study*, contains task‐based EEG recordings from 43 participants (19 patients with psychosis and 24 healthy controls) (19). All data were collected at the University of Fribourg, Switzerland, in compliance with ethical approval (Ref: 054/13‐CER‐FR) and are shared in the BIDS format. The analyses presented in this manuscript were conducted as a secondary data analysis using this publicly available dataset.
